# Author Correction: 3D-printed liquid metal polymer composites as NIR-responsive 4D printing soft robot

**DOI:** 10.1038/s41467-024-45465-y

**Published:** 2024-01-31

**Authors:** Liwen Zhang, Xumin Huang, Tim Cole, Hongda Lu, Jiangyu Hang, Weihua Li, Shi-Yang Tang, Cyrille Boyer, Thomas P. Davis, Ruirui Qiao

**Affiliations:** 1 Australian Institute of Bioengineering & Nanotechnology, The University of Queensland, Brisbane, QLD 4072 Australia; 2 Department of Electronic, Electrical, and Systems Engineering, University of Birmingham, Birmingham, UK; 3 School of Mechanical, Materials, Mechatronic and Biomedical Engineering, University of Wollongong, Wollongong, NSW 2522 Australia; 4 School of Electronics and Computer Science, University of Southampton, Southampton, SO17 1BJ UK; 5 Cluster for Advanced Macromolecular Design, School of Chemical Engineering, The University of New South Wales, Sydney, NSW 2052 Australia

**Keywords:** Polymers, Polymers, Actuators

Correction to: *Nature Communications* 10.1038/s41467-023-43667-4, published online 28 November 2023

The original version of this Article contained an error in Fig. 2a, in which the molecular structure of tert-butyl acrylate (TBAm) is incorrect. This has been corrected in both the PDF and HTML versions of the Article.

