# Supplementary material for: 3D-printed liquid metal polymer composites as NIR-responsive 4D printing soft robot
Source: Nat Commun. 2023 Nov 28;14:7815. doi: 10.1038/s41467-023-43667-4 (PMC10684855; doi:10.1038/s41467-023-43667-4)
Supplement: Supplementary file 1 — Supplementary Information [file 41467_2023_43667_MOESM1_ESM.pdf]

Supplementary Information

3D-Printed Liquid Metal Polymer Composites as NIR-Responsive 4D Printing Soft Robot

Liwen Zhang[1], Xumin Huang[1], Tim Cole[2], Hongda Lu[1,3], Jiangyu Hang[1], Weihua Li[3], Shi-Yang Tang[4,], Cyrille Boyer[5], Thomas P. Davis[1,*], Ruirui Qiao[1,*]*

[1]Australian Institute of Bioengineering & Nanotechnology, The University of Queensland, Brisbane, Queensland 4072, Australia.

[2]Department of Electronic, Electrical, and Systems Engineering, University of Birmingham, Birmingham, UK.

[c]School of Mechanical, Materials, Mechatronic and Biomedical Engineering, University of Wollongong, Wollongong, NSW 2522, Australia.

[4]School of Electronics & Computer Science, University of Southampton, Southampton, SO17 1BJ, UK.

[5]Cluster for Advanced Macromolecular Design and School of Chemical Engineering, The University of New South Wales, Sydney, NSW 2052 Australia

[*]Corresponding authors, Email: shiyang.tang@soton.ac.uk, t.davis@uq.edu.au, r.qiao@uq.edu.au

Supplementary Notes: Tables

Supplementary Table 1. The mechanical properties of 3D-printed LMPCs[a]

#	RLMNPs	T_g (°C)	G' (MPa)[b]	σ (MPa)	*E* (MPa)
1	0 wt%	45.65 ± 0.75	2645.34 ± 57.96	18.00 ± 1.76	434.38 ± 20.63
2	0.5 wt%	43.45 ± 0.72	2545.50 ± 71.74	17.34 ± 0.55	388.69 ± 5.93
3	1 wt%	40.54 ± 0.71	1989.39 ± 49.44	13.22 ± 0.31	318.06 ± 11.21
4	2 wt%	39.31 ± 0.87	1846.92 ± 35.84	6.37 ± 0.72	173.88 ± 13.95

[a] Experimental conditions: [RAFT]: [TPO]: [TBAm+PEGDA] = 1:2.5:200, a mass ratio of [TBAm]:[PEGDA] = 65:35. 3D-printed objects were fabricated by a commercially available SLA 3D printer under light-emitting diode (LED) irradiation (λ_{max} = 405 nm, 0.81 mW cm^{-2}); T_g, G', σ, and *E* represents glass transition temperature, storage modulus, tensile stress, and young's modulus, respectively. [b] G' of 3D-printed objects was determined at 20 °C using DMA. Data were expressed as means ± SD (n = 3 independent 3D-printed samples).

Supplementary Table 2. The mechanical properties of 3D-printed LMPCs with different monomers[a]

#	Monomer	T_g (°C)	G' (MPa)[b]	σ (MPa)	*E* (MPa)
1	HEAAm	70.12 ± 1.46	4190.48 ± 24.27	46.77 ± 3.11	603.57 ± 14.57
2	DMAm	44.13 ± 0.21	2161.98 ± 30.19	18.51 ± 0.19	288.45 ± 14.57
3	TBAm	40.42 ± 0.25	1942.74 ± 17.88	17.41 ± 0.91	323.32 ± 29.34
4	HEAm	18.33 ± 0.52	173.80 ± 4.02	2.55 ± 0.68	15.27 ± 0.34

[a] Experimental conditions: [RAFT]: [TPO]: [M+PEGDA] = 1:2.5:200, a mass ratio of [M]:[PEGDA] = 65:35, and 0.5 wt% of RLMNPs in 3D-printed materials. LMPCs were fabricated by a commercially available SLA 3D printer under light-emitting diode (LED) irradiation (λ_{max} = 405 nm, 0.81 mW cm^{-2}) using HEAAm, DMAm, HEAm, and TBAm as monomers; T_g, G', σ, and *E* represents glass transition temperature, storage modulus, tensile stress, and young's modulus, respectively. [b] The storage modulus of 3D-printed objects was determined at 20 °C using DMA. Data were expressed as means ± SD (n = 3 independent 3D-printed samples).

Supplementary Table 3. The mechanical properties of 3D-printed LMPCs with different mass ratios of [TBAm]: [PEGDA][a]

#	[TBAm]: [PEGDA]	T_g (°C)	G' (MPa)[b]	σ (MPa)	*E* (MPa)
1	0:100	60.30 ± 2.19	2723.72 ± 35.25	23.66 ± 1.39	404.27 ± 7.64
2	35:65	49.74 ± 0.48	2677.09 ± 30.26	16.64 ± 1.07	365.55 ± 28.99
3	65:35	40.73 ± 0.56	1969.94 ± 28.76	14.98 ± 0.71	315.93 ± 17.94

[a] Experimental conditions: [RAFT]: [TPO]: [M+PEGDA] = 1:2.5:200 and 0.5 wt% of RLMNPs in 3D-printed materials. LMPCs were fabricated by a commercially available SLA 3D printer under light-emitting diode (LED) irradiation (λ_{max} = 405 nm, 0.81 mW cm^{-2}); T_g, G', σ, and *E* represents glass transition temperature, storage modulus, tensile stress, and young's modulus, respectively. [b] The storage modulus of 3D-printed objects was determined at 20 °C using DMA. Data were expressed as means ± SD (n = 3 independent 3D-printed samples).

Supplementary Notes: Figures

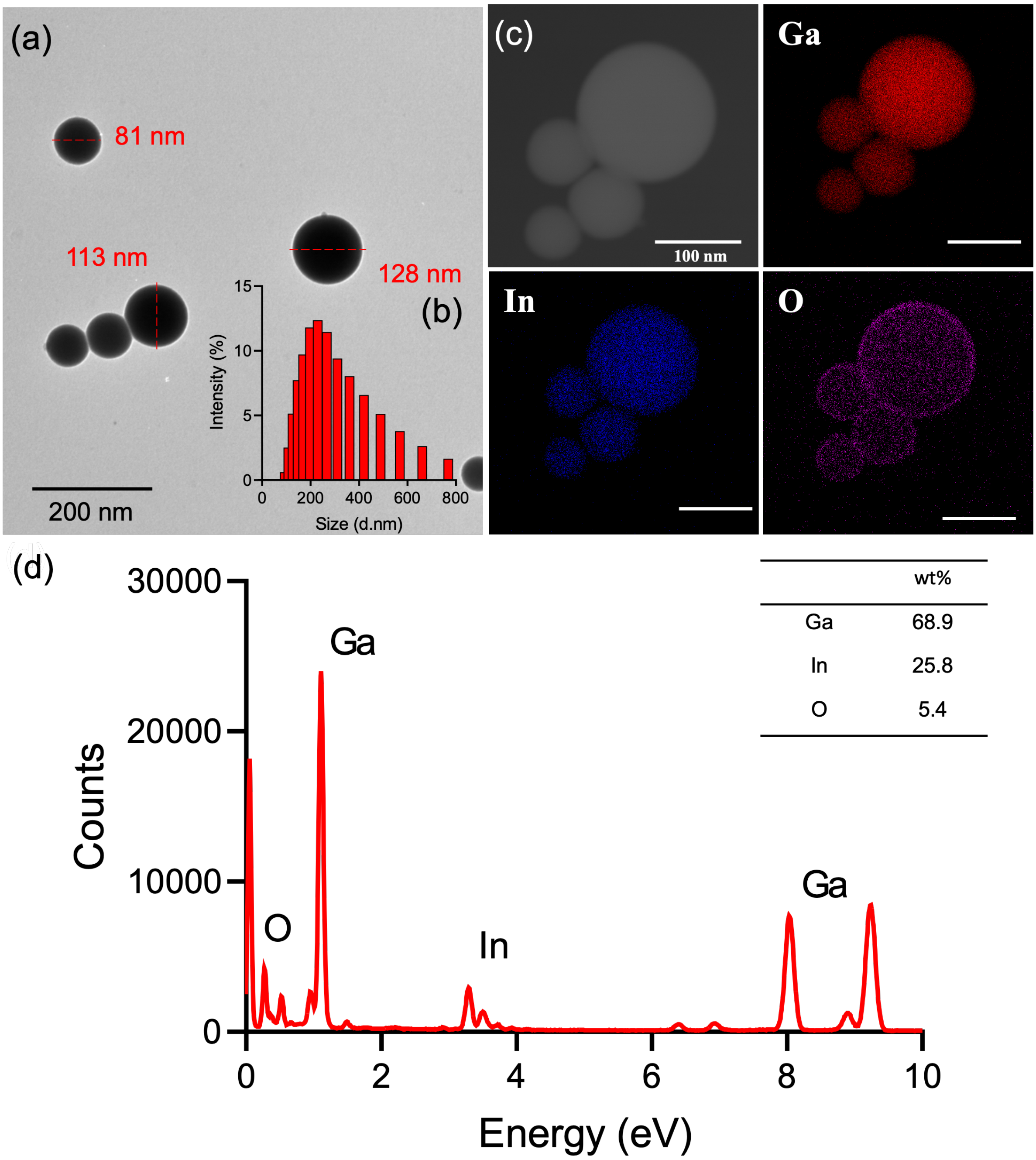

Supplementary Fig. 1. The characterization of LMNPs. (a) The TEM photograph of LMNPs; (b) The insert exhibits intensity-based size distribution histograms of LMNPs measured by DLS; (c) The elemental mapping and (d) EDS analysis of LMNPs including Ga, In, and O elements. The experiments were repeated independently three times with similar results.

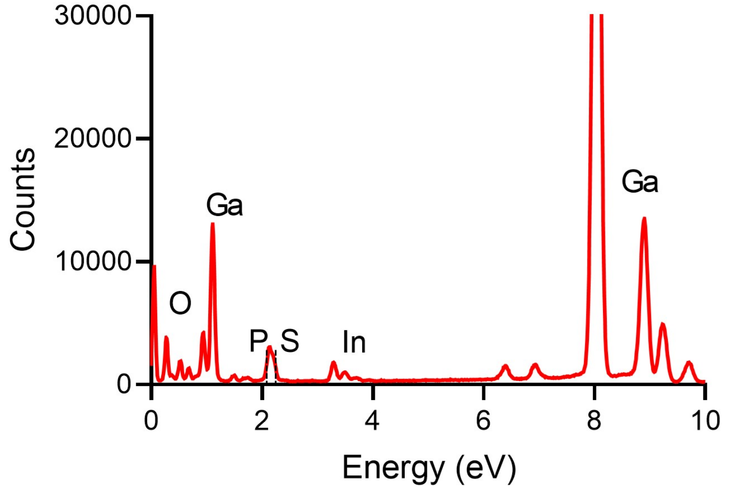

Supplementary Fig. 2. EDS analysis of RLMNPs including Ga, In, O, and P elements.

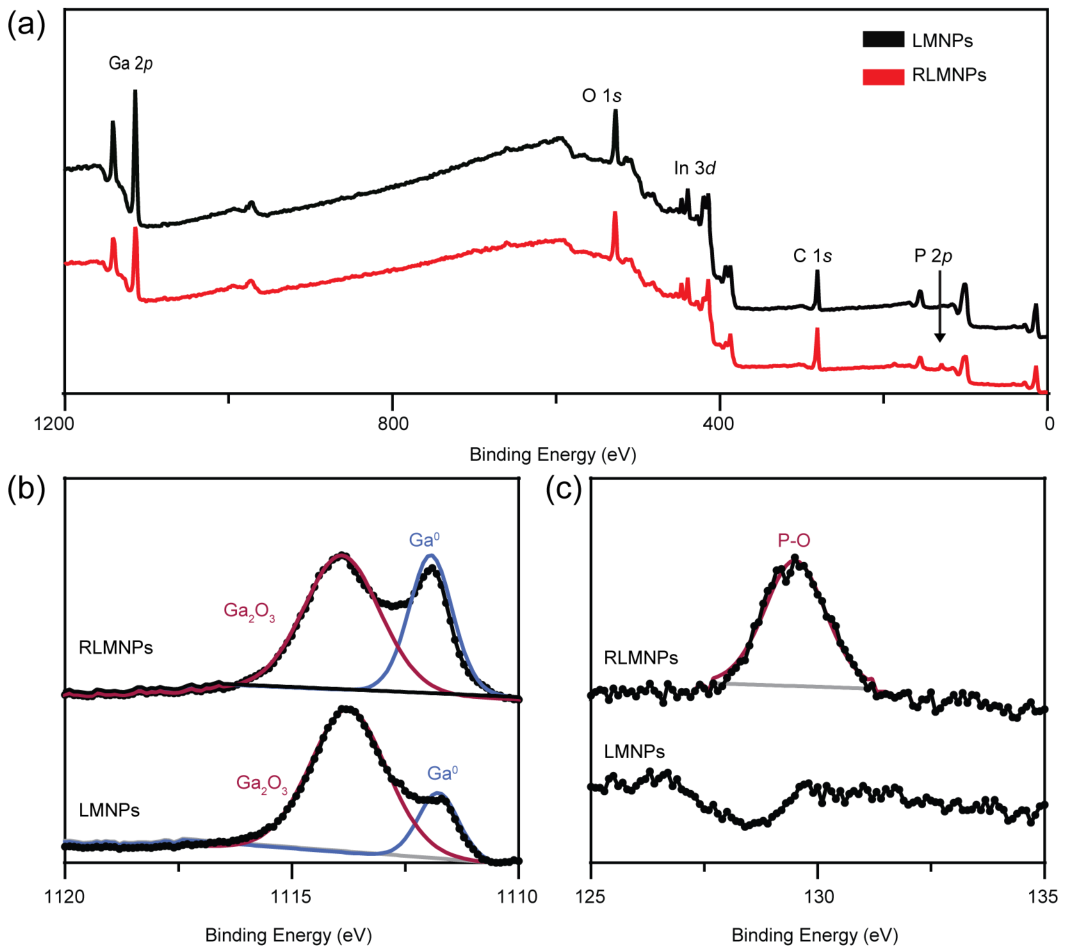

Supplementary Fig. 3. The XPS spectrum of liquid metal nanoparticles (LMNPs) and reversible addition−fragmentation chain-transfer agents grafted LMNPs (RLMNPs). (a) a typical XPS spectrum, and a high-resolution XPS of (b) Ga 2*p*3/2 and (c) P 2*p*.

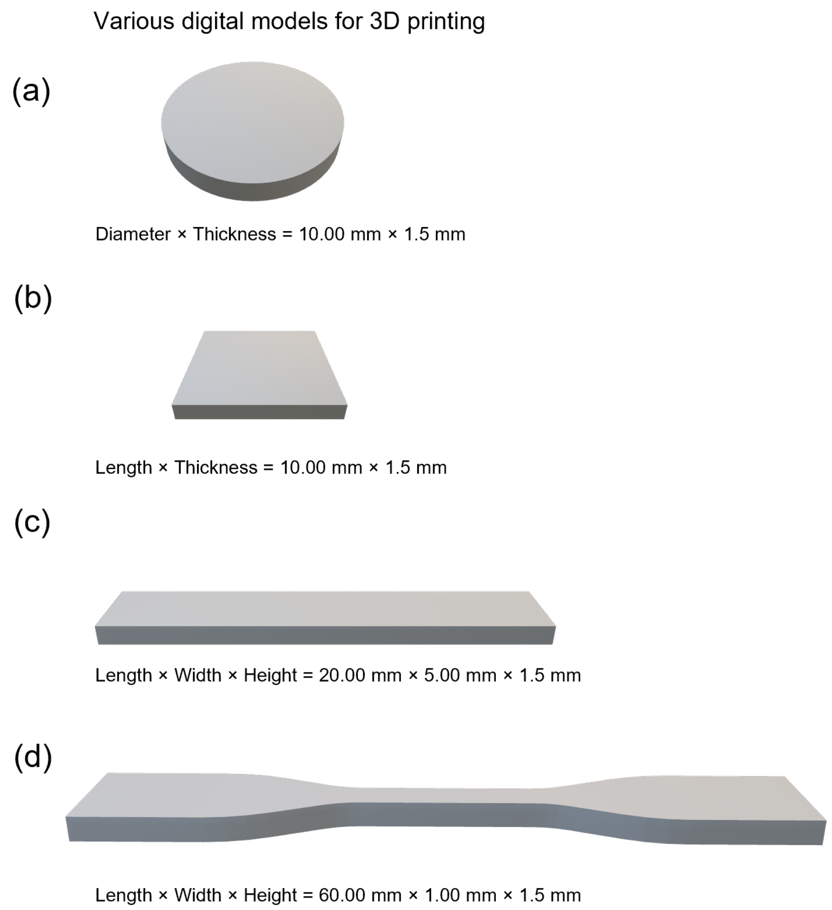

Supplementary Fig. 4. Various digital models for 3D printing, including (a) cylinder, (b) (c) cubid, and (d) standard specimens for tensile test.

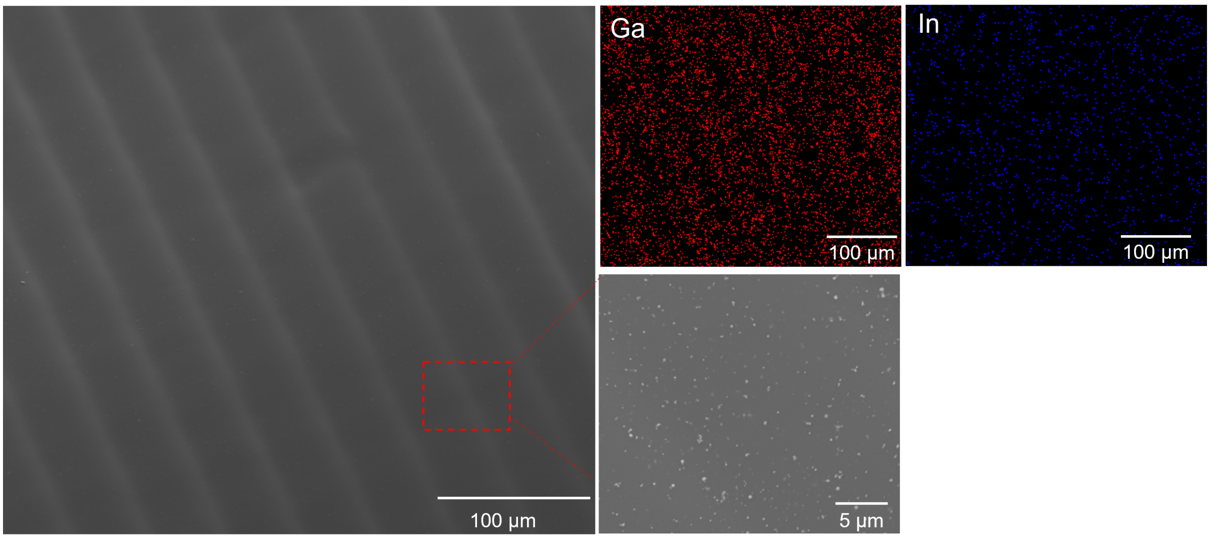

Supplementary Fig. 5. The SEM photograph and EDS elemental mapping of LMPCs without RAFT agents; the detected elements included Ga and In. The experiments were repeated independently three times with similar results.

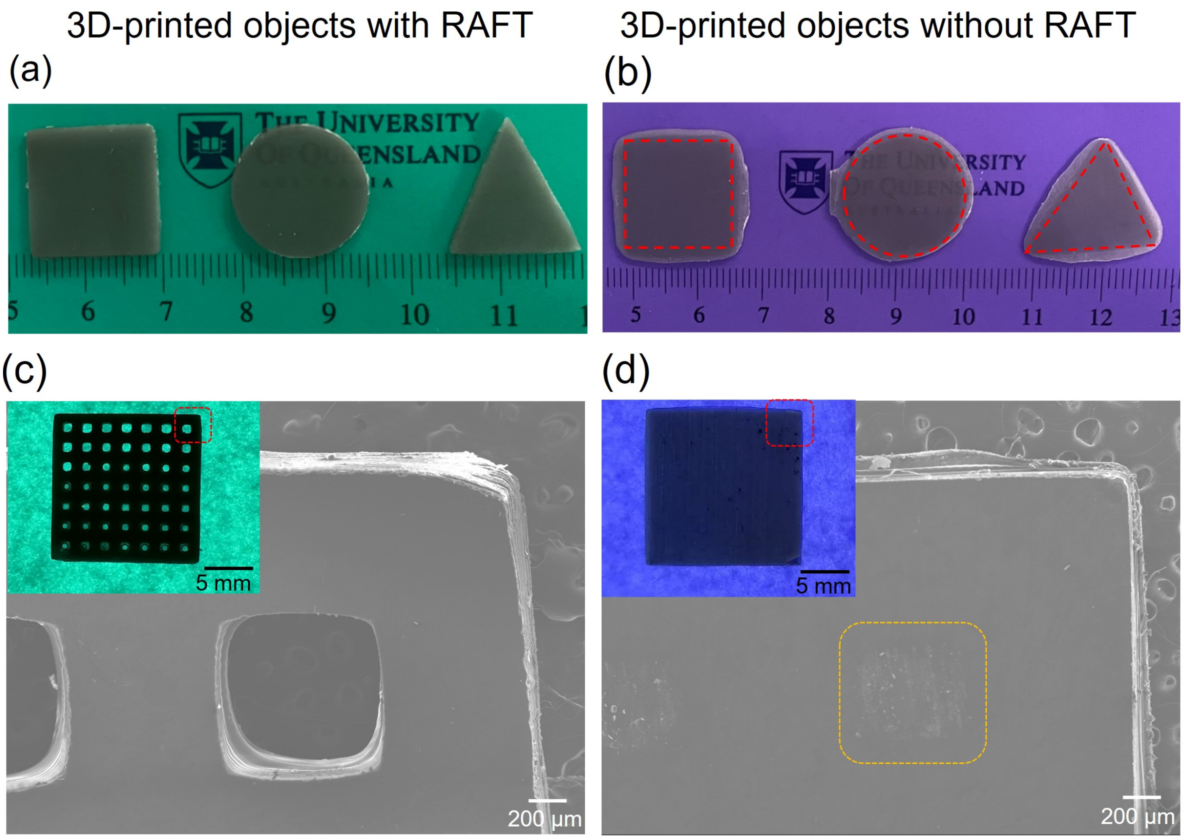

Supplementary Fig. 6. Resolution comparison of 3D-printed objects with and without reversible addition−fragmentation chain-transfer (RAFT) agents. (a) Photo of 3D-printed objects with RAFT agents using a layer cure time of 40 s; (b) Photo of the RAFT-free 3D-printed object under an identical condition; (c) SEM image of the 3D-printed porous object with RAFT agents. The insert on the upper left displays a photo of the 3D-printed porous object with RAFT agents; (d) SEM image of the RAFT-free 3D-printed porous object. The insert on the upper left displays a photo of the RAFT-free 3D-printed porous object. The experiments were repeated independently three times with similar results.

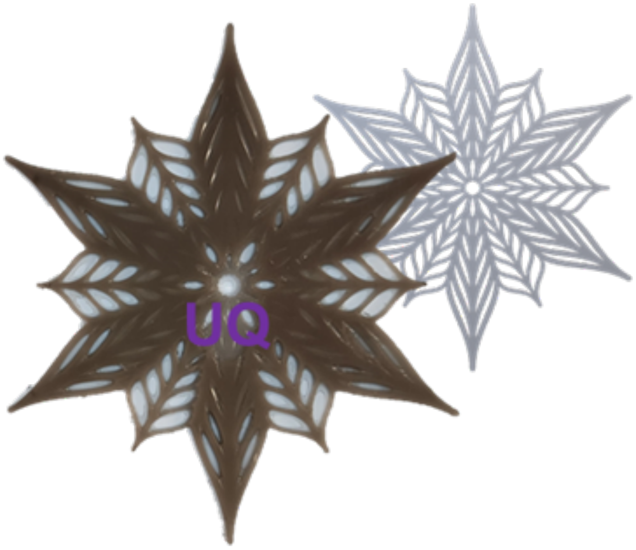

Supplementary Fig. 7. The snowflake-like composite was fabricated by a stereolithography 3D printer. The grey model represents the digital model.

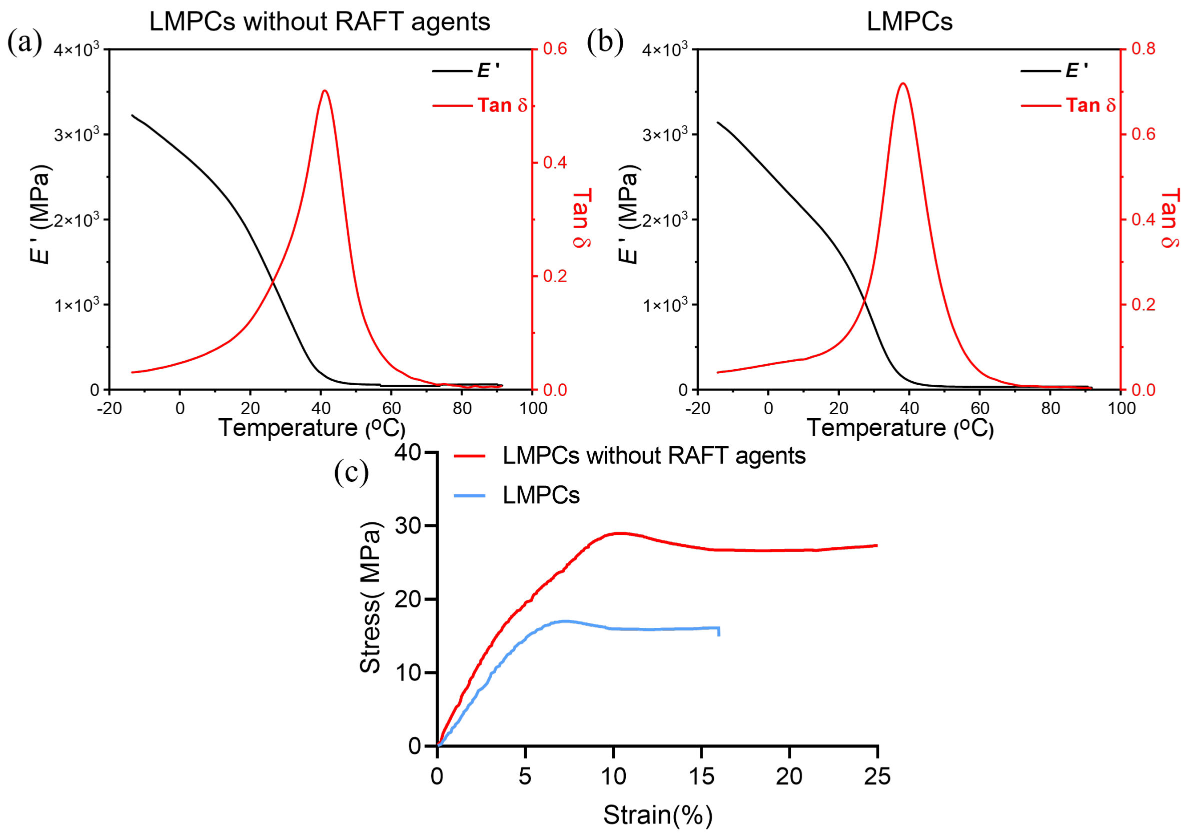

Supplementary Fig. 8. The mechanical properties testing of 3D-printed liquid metal polymer composites (LMPCs). (a) Storage modulus (E′) and Tan δ of the 3D-printed LMPCs without reversible addition−fragmentation chain-transfer (RAFT) agents at a frequency of 1 Hz; (b) Storage modulus (E′) and Tan δ of the 3D-printed object with LMPCs at a frequency of 1 Hz; (c) Tensile tests of 3D-printed objects that contained LMNPs and RLMNPs, respectively.

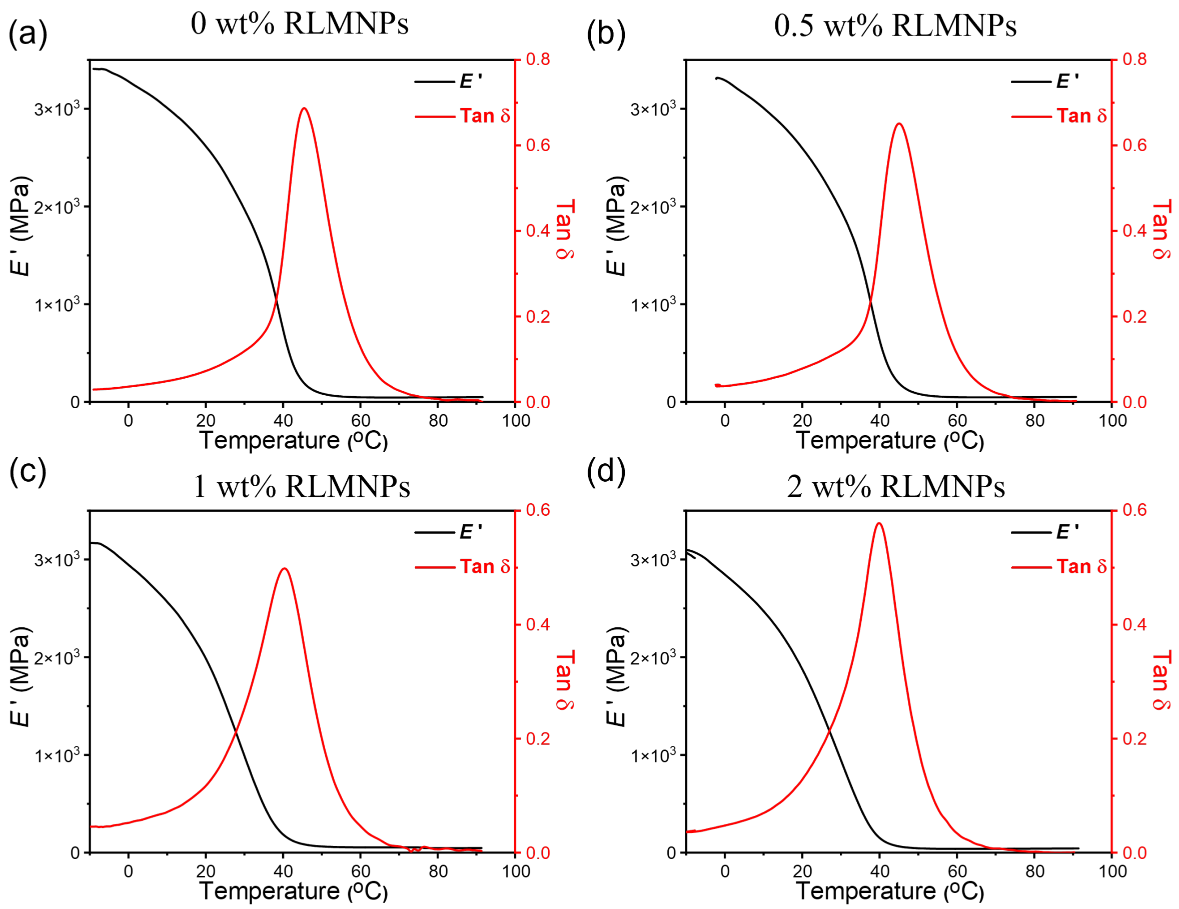

Supplementary Fig. 9. The DMA of 3D-printed LMPCs with different concentrations of reversible addition−fragmentation chain-transfer agents grafted liquid metal nanoparticles (RLMNPs). Storage modulus (E′) and Tan δ of 3D-printed objects with (a) 0 wt%, (b) 0.5 wt%, (c) 1 wt%, and (d) 2 wt% RLMNPs at a frequency of 1 Hz.

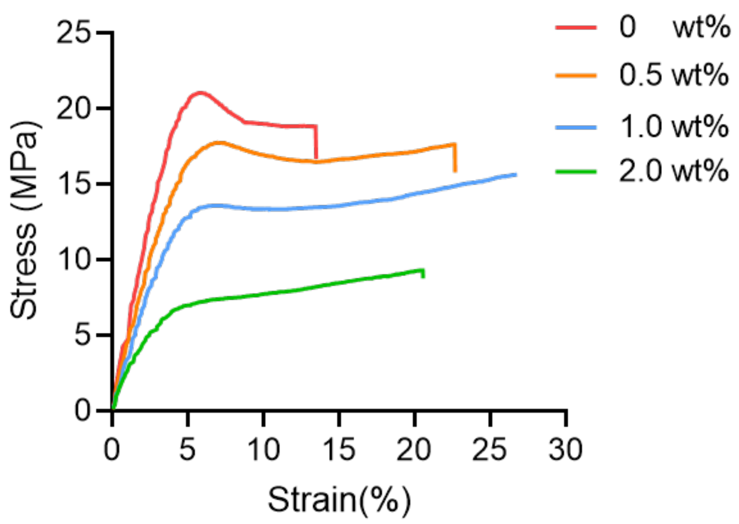

Supplementary Fig. 10. Tensile tests of 3D-printed LMPCs containing different concentrations of RLMNPs (0, 0.5, 1, and 2 wt%).

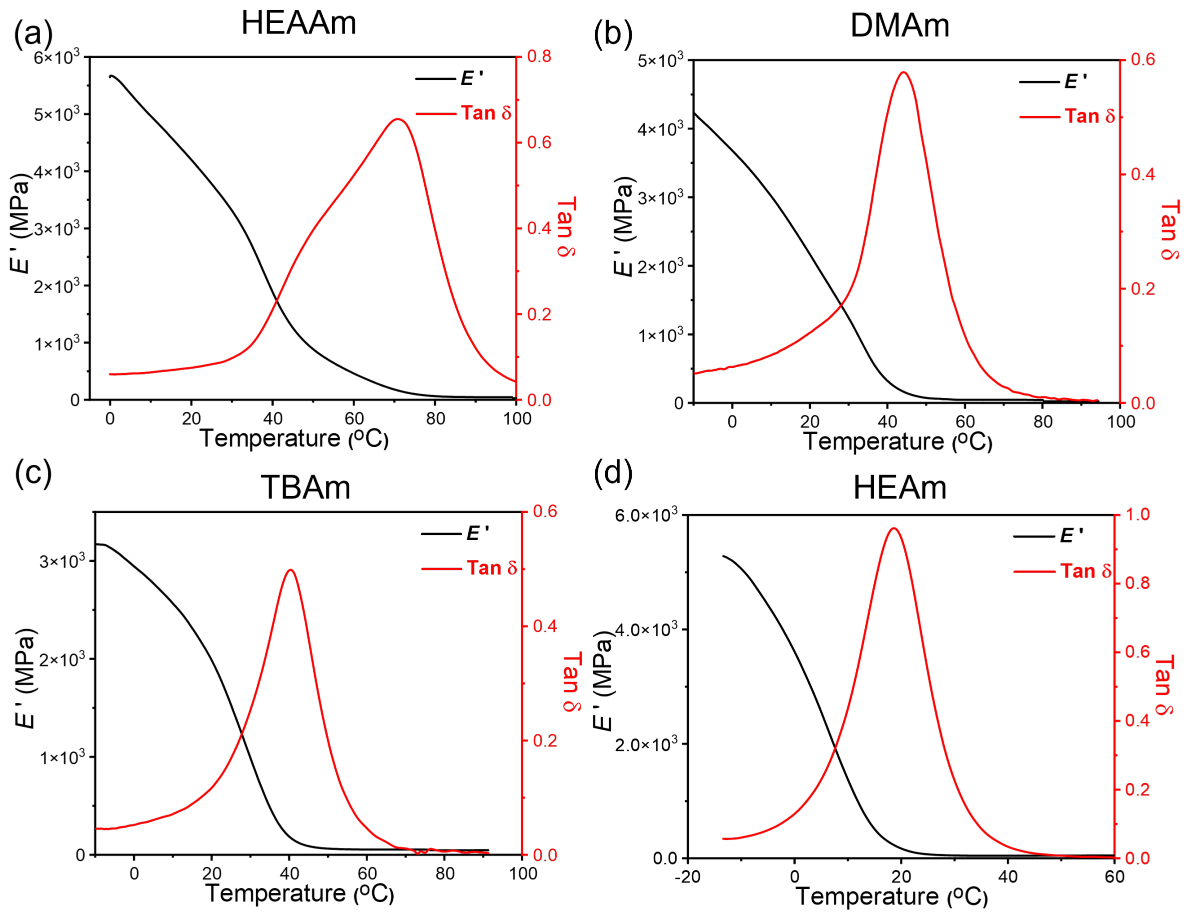

Supplementary Fig. 11. The dynamic mechanical analysis of 3D-printed LMPCs using (a) N-hydroxyethyl acrylamide (HEAAm), (b) N, N-dimethyl acrylamide (DMAm), (c) *tert*-butyl acrylate (TBAm), and (d) 2-hydroxyethyl acrylate (HEAm) as model monomers with a fixed molar ratio of [RAFT]:[TPO]:[TBAm+PEGDA] = 1: 2.5: 200 and a wt% of [RLMNPs]: [M]: [PEGDA] = 1: 65: 35.

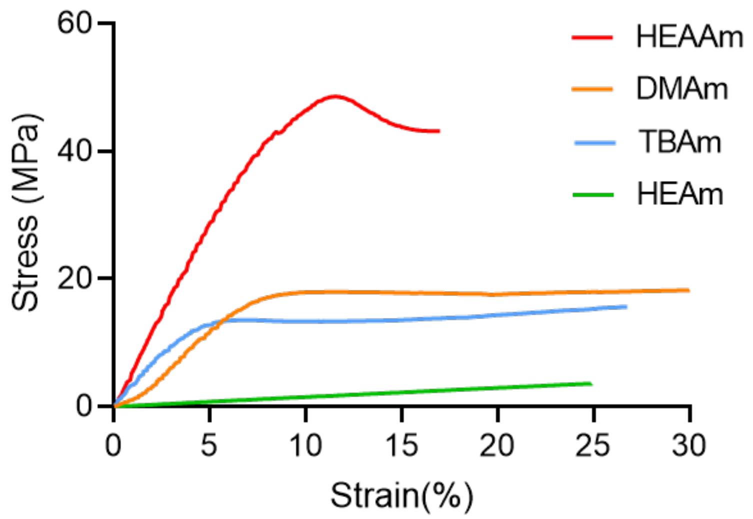

Supplementary Fig. 12. Tensile tests of 3D-printed LMPCs using different monomers.

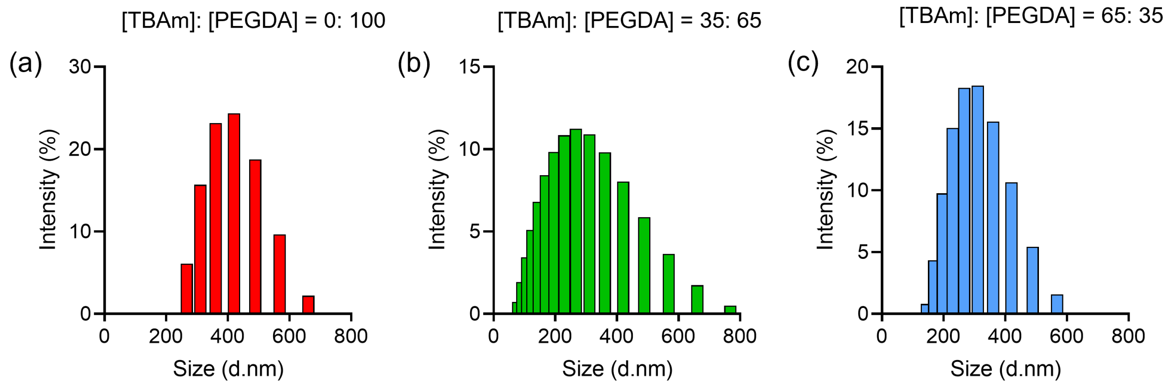

Supplementary Fig. 13. DLS intensity-based size distribution histograms of RLMNPs in the mixed ink with different percentages of the weight of [*tert*-butyl acrylate (TBAm)] and [poly(ethylene glycol) diacrylate (PEGDA)], including (a) [TBAm]: [PEGDA] = 0: 100, (b) [TBAm]: [PEGDA] = 35: 65, and (c) [TBAm]: [PEGDA] = 65: 35.

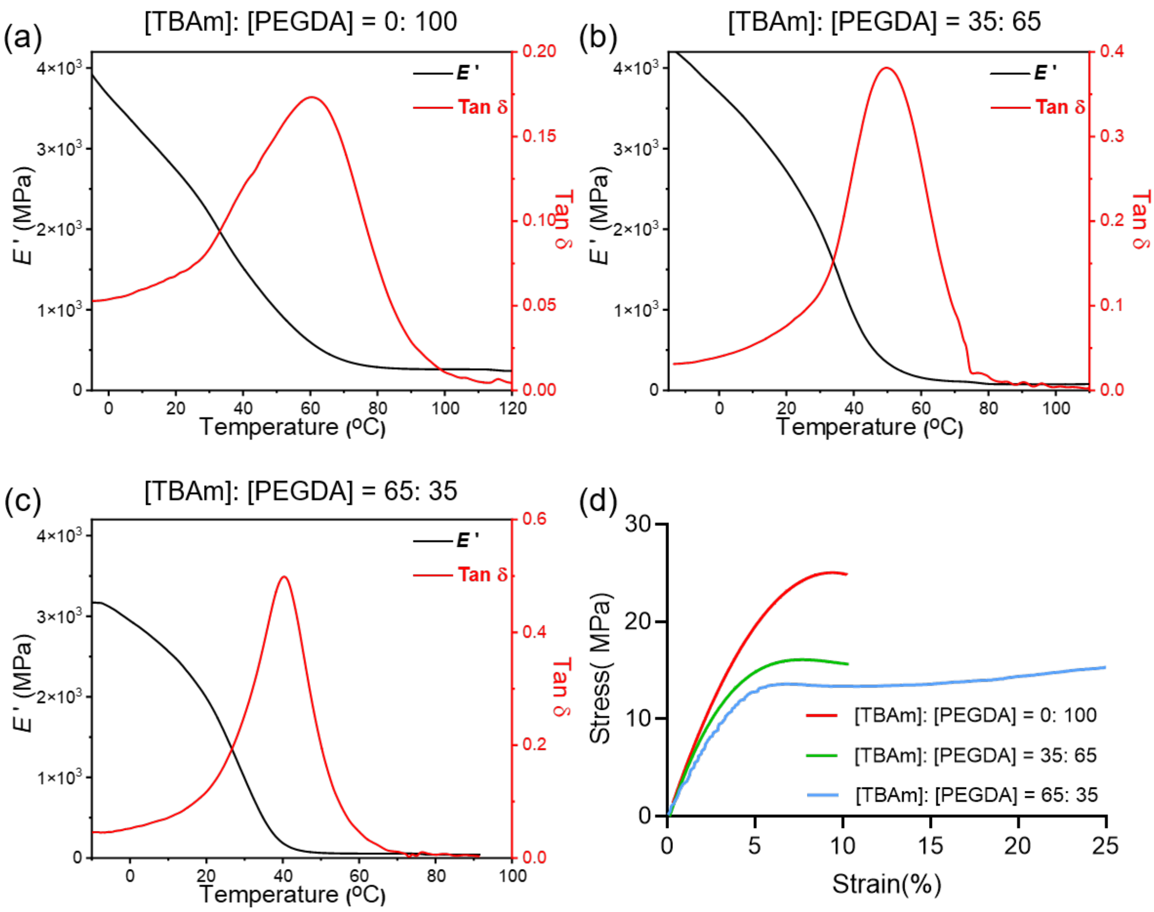

Supplementary Fig. 14. The mechanical properties testing of 3D-printed LMPCs with different percentages of the weight (wt%) of [*tert*-butyl acrylate (TBAm)] and [poly(ethylene glycol) diacrylate (PEGDA)]. Storage modulus (E′) and Tan δ of 3D-printed objects were determined using different wt% of monomers and crosslinker as 3D printing ink, including (a) [TBAm]: [PEGDA] = 0: 100, (b) [TBAm]: [PEGDA] = 35: 65, and (c) [TBAm]: [PEGDA] = 65: 35; (d) Tensile tests of 3D-printed objects with different ratio of [TBAm] and [PEGDA].

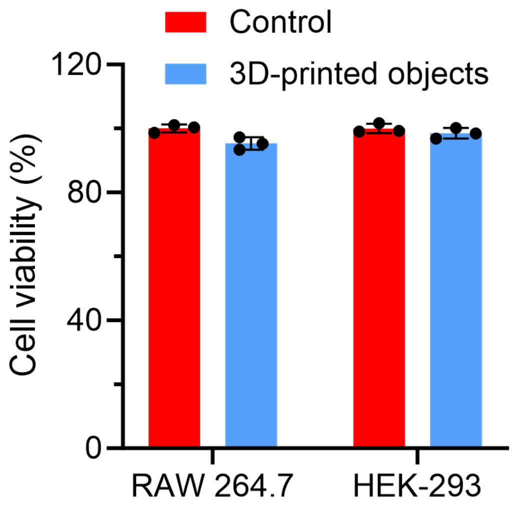

Supplementary Fig. 15. Cell viability studies with and without 3D-printed objects on RAW-264.7 and HEK-293 cells. Bars represent means ± SD (n = 3 independent cell experiments).

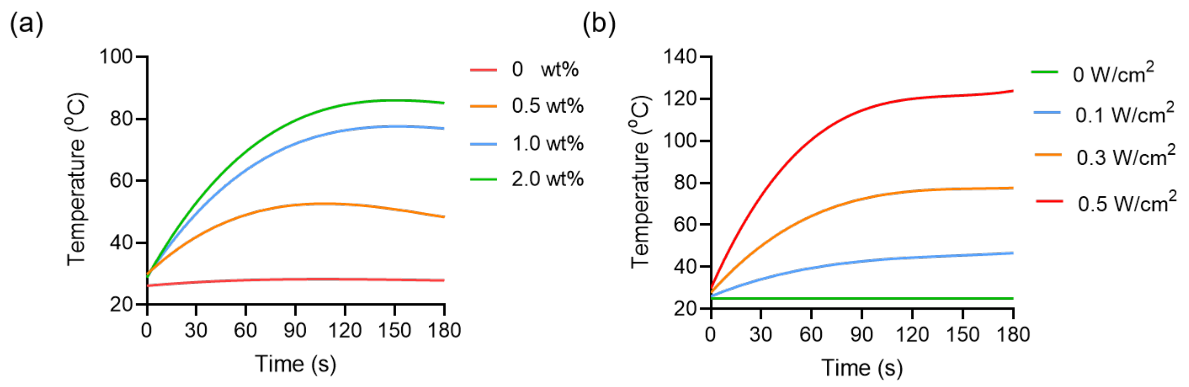

Supplementary Fig. 16. (a) Photothermal effects of 0.3W/ cm^2 808 nm laser with 0, 0.5, 1.0, and 2.0 wt% of RLMNPs; (b) 808 nm laser with different light intensity (0, 0.1, 0.3, and 0.5 W/cm^2) heating LMPCs with 1 wt% RLMNPs, ambient temperature: 25 °C.

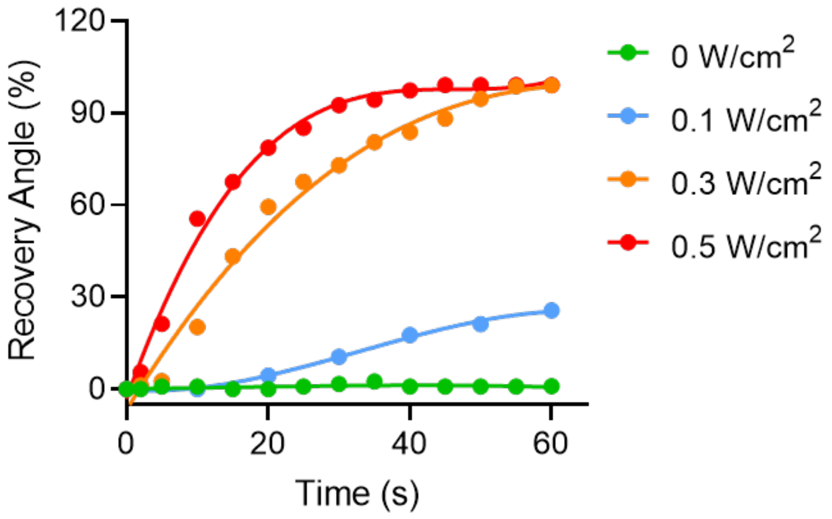

Supplementary Fig. 17. The recovery angle on the time of 3D-printed LMPCs under NIR irradiation (λ_{max}= 808 nm) with different light intensities from 0 to 0.5 W/cm^2

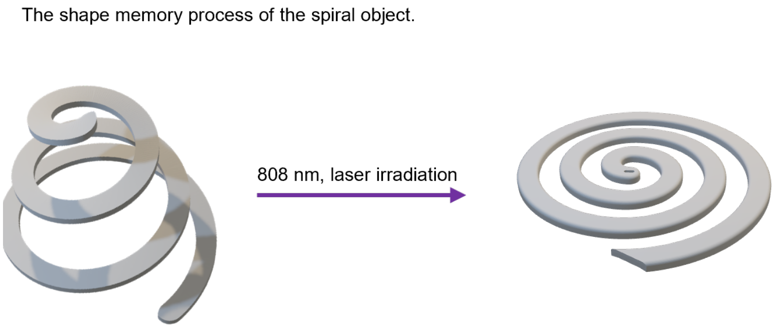

Supplementary Fig. 18. The digital model represents the shape memory process of the spiral object.

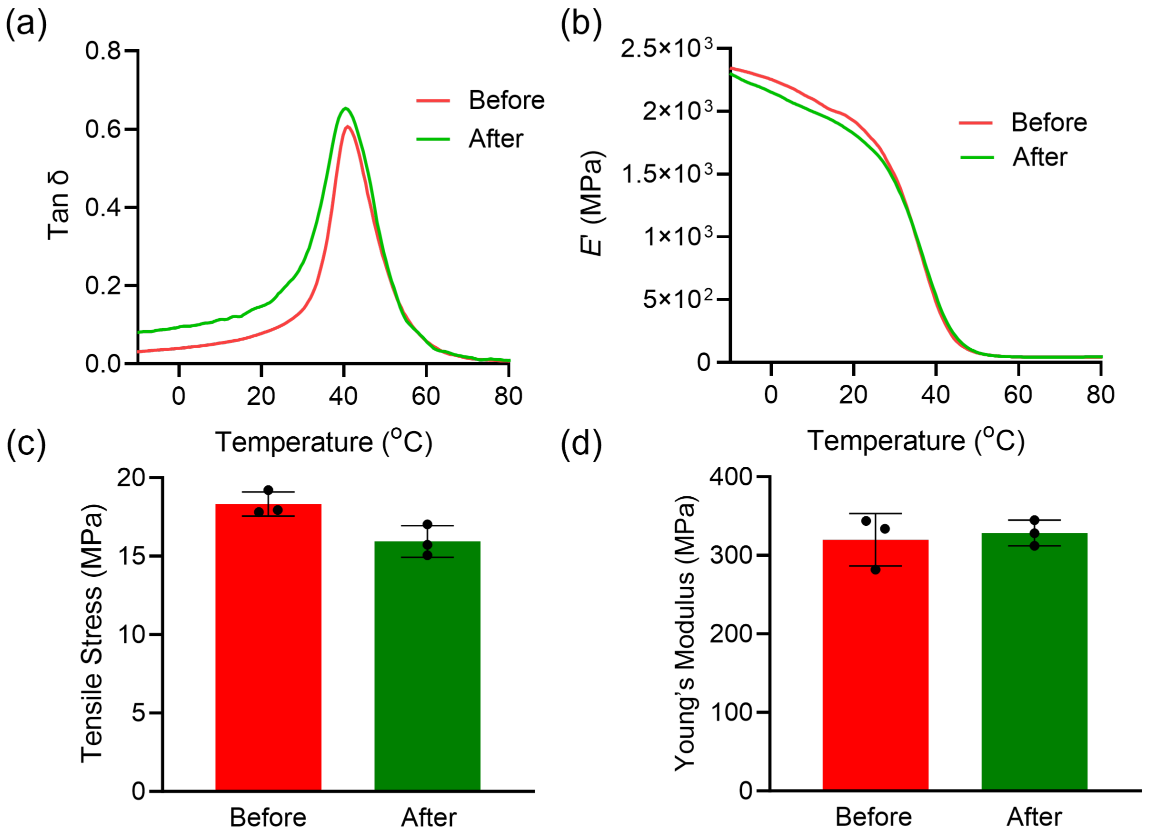

Supplementary Fig. 19. The mechanical properties testing of LMPCs before and after performing 4D printing. (a) Tan δ, (b) Storage modulus (E′), (c) Tensile Stress, and (d) Young's modulus of 3D-printed objects before and after performing the shape memory process. Bars in (c and d) represent means ± SE (n = 3 independent 3D-printed objects).

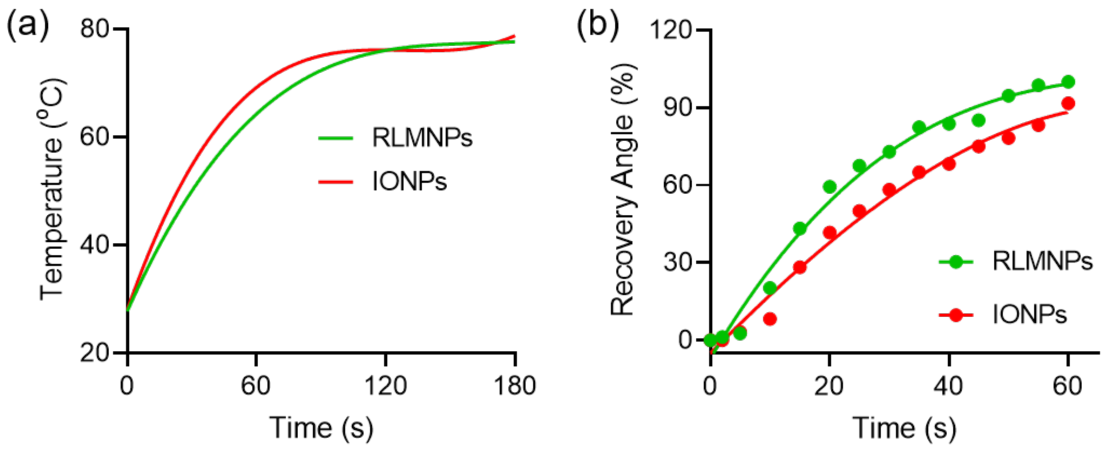

Supplementary Fig. 20. (a) 0.3 W/cm^2 laser heating 3D-printed materials that contained reversible addition−fragmentation chain-transfer agents grafted liquid metal nanoparticles (RLMNPs) and iron oxide nanoparticles (IONPs), respectively, ambient temperature: 25 °C; (b) recovery curves on time of 3D-printed materials that respectively containing RLMNPs and IONPs while irradiating with 808 nm laser (0.3 W/cm^2) for 60 s.

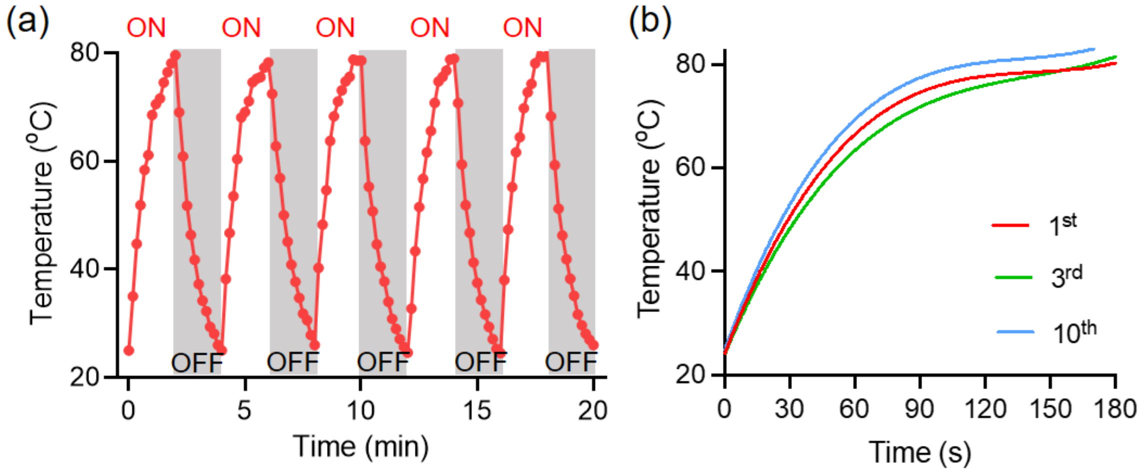

Supplementary Fig. 21. (a) Temperature variation of 3D-printed LMPCs under NIR irradiation irradiation (λ_{max}= 808 nm, 0.3 W/cm^2) for 5 on/off cycles (2 min irradiation for each cycle); (b) Photothermal effect of 3D-printed LMPCs at the 1, 3, and 10th cycles.

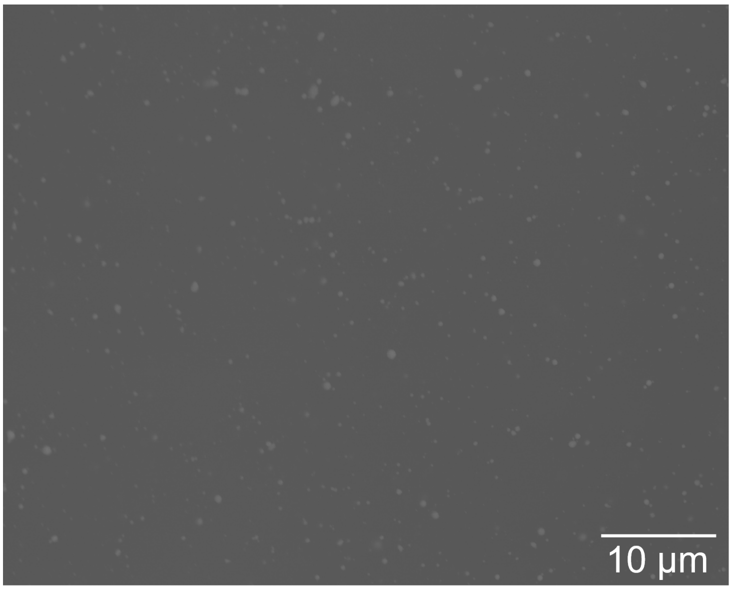

Supplementary Fig. 22. The SEM photograph of the deformed part of 3D-printed LMPCs after 25 shape memory cycles. The experiments were repeated independently three times with similar results.

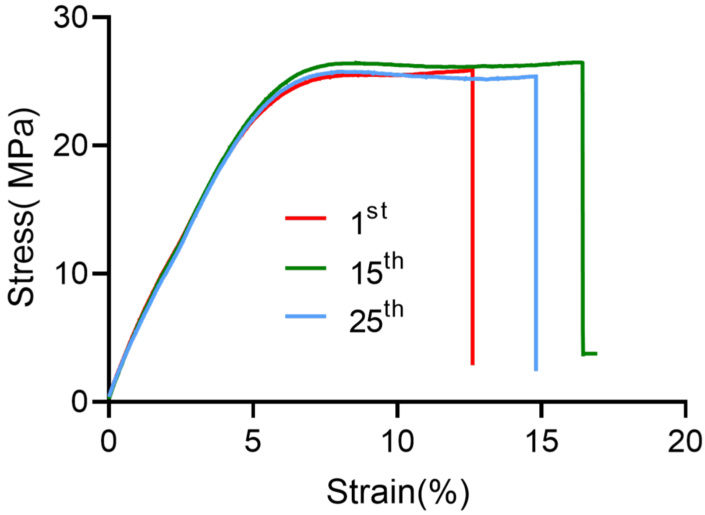

Supplementary Fig. 23. Tensile tests of 3D printed objects in a total of 25 cycles.

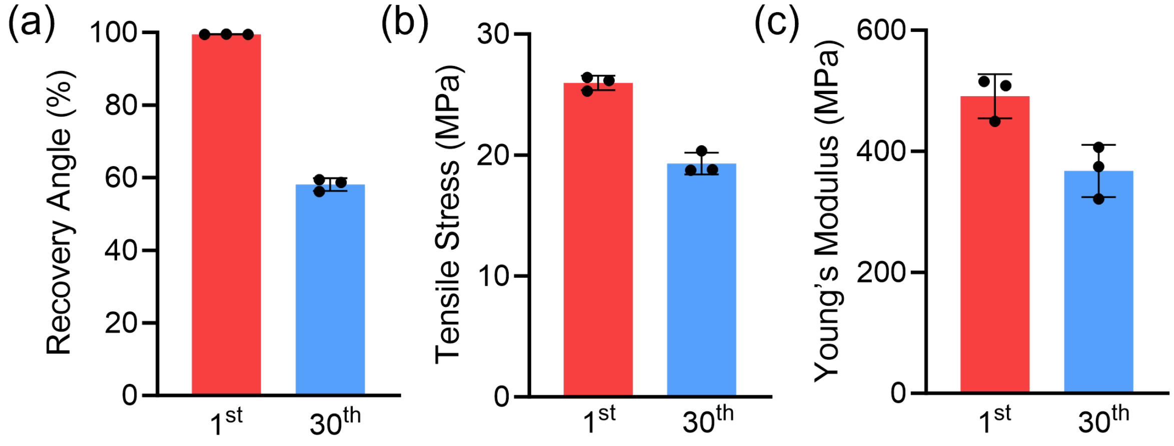

Supplementary Fig. 24. (a) Recovery Angle of LMPCs at the first and 30th cycle of 4D printing when irradiation with NIR light for 60 seconds; (b) Tensile stress, and (c) Youngs' modulus of LMPCs at the first and 30th cycle of 4D printing. Bars represent means ± SE (n = 3 independent 3D-printed objects).
