# Supplementary material for: 3D-printed liquid metal polymer composites as NIR-responsive 4D printing soft robot
Source: Nat Commun. 2023 Nov 28;14:7815. doi: 10.1038/s41467-023-43667-4 (PMC10684855; doi:10.1038/s41467-023-43667-4)
Supplement: Supplementary file 3 — Description of Additional Supplementary Files [file 41467_2023_43667_MOESM3_ESM.pdf]

Description of additional supplementary files

Supplementary Movie 1. NIR-mediated shape recovery of LMPCs.

Supplementary Movie 2. NIR-responsive LMPCs for controlled object release.

Supplementary Movie 3. NIR light-responsive LMPCs for gripping and releasing an object
